# Investigating a cluster of vulvar cancer in young women: a cross-sectional study of genital human papillomavirus prevalence

**DOI:** 10.1186/1471-2334-12-243

**Published:** 2012-10-05

**Authors:** Alice R Rumbold, Sarah E Tan, John R Condon, Debbie Taylor-Thomson, Maria Nickels, Sepehr N Tabrizi, Margaret LJ Davy, Margaret M O’Brien, Christine M Connors, Ibrahim Zardawi, Jim Stankovich, Suzanne M Garland

**Affiliations:** 1 Discipline of Obstetrics & Gynaecology, The University of Adelaide, Adelaide, SA 5005, Australia; 2 Epidemiology and Health Systems Division, Menzies School of Health Research, PO Box 41096, Casuarina, NT 0811, Australia; 3 Clinical Microbiology and Infectious Diseases, Royal Women’s Hospital, and Department of Obstetrics and Gynaecology, University of Melbourne, Royal Women’s Hospital, Cnr of Flemington Road and Grattan Street, Parkville, VIC 3052, Australia; 4 WHO HPV LabNet Regional Reference Laboratory - Western Pacific Region, Melbourne, VIC, Australia; 5 Health Services Division, Northern Territory Department of Health, PO Box 40596, Casuarina, NT 0811, Australia; 6 Infectious Diseases and Microbiology Group, Murdoch Children’s Research Institute, Bio 21 Institute, Level 1 Building 404, 30 Flemington Rd, Parkville, Victoria 3052, Australia; 7 Surgical and Specialties Service, Royal Adelaide Hospital, North Tce, Adelaide, SA 5000, Australia; 8 Department of Obstetrics and Gynaecology, Cairns Base Hospital, The Esplanade, Cairns, QLD 4870, Australia; 9 Discipline of Anatomical Pathology, University of Newcastle, Manning Health Campus, PO Box 649, Taree, NSW, 2430, Australia; 10 Biostatistics Group, Menzies Research Institute Tasmania, University of Tasmania, Medical Sciences Building 1, 17 Liverpool Street, Private Bag 23, Hobart, TAS 7000, Australia

**Keywords:** Human papillomavirus, Population prevalence, Vulvar neoplasms, Young women, Indigenous women

## Abstract

**Background:**

Vulvar cancer is a relatively rare malignancy, which occurs most often in postmenopausal women. We have previously identified a geographic cluster of vulvar cancer in young Indigenous women living in remote communities in the Arnhem Land region of Australia. In this population, we investigated the prevalence of oncogenic human papillomavirus (HPV) infection in anogenital samples (vulvar/vaginal/perianal area and cervix) and compared the overall, type-specific and multiple infection prevalence between sites.

**Methods:**

A cross-sectional survey of 551 Indigenous women aged 18–60 years was undertaken in 9 Arnhem Land communities. Women were consented for HPV detection and genotyping collected by a combined vulvar/vaginal/perianal (VVP) sweep swab and a separate PreservCyt endocervical sample collected during Pap cytology screening. HPV DNA testing was undertaken using PCR with broad spectrum L1 consensus PGMY09/11 primers with genotyping of positive samples by Roche Linear Array. The primary outcomes were the prevalence of cervical and VVP high-risk (HR) HPV.

**Results:**

The prevalence of VVP HR-HPV was 39%, which was significantly higher than the cervical HR-HPV prevalence (26%, p<0.0001). HPV-16 was the most common genotype detected in both sites (VVP 11%, cervical 6%). HPV-16 infection peaked in women aged <20 years; however, there was a marked decline in cervical HPV-16 prevalence with age (p=0.007), whereas following an initial decline, the prevalence of VVP HPV-16 remained constant in subsequent age-groups (p=0.835).

**Conclusions:**

In this population experiencing a cluster of vulvar cancer, the prevalence of cervical oncogenic HPV infection was similar to that reported by studies of other Australian women; however there was a significantly higher prevalence of vulvar/vaginal/perianal infection to cervical. The large discrepancy in HPV prevalence between anogenital sites in this population may represent more persistent infection at the vulva. This needs further investigation, including the presence of possible environmental and/or genetic factors that may impair host immunity.

## Background

Vulvar cancer is a relatively rare malignancy, accounting for approximately 3% of all gynecological cancers worldwide [1]. Two independent etiological pathways have been implicated in the development of vulvar cancer. In younger women, persistent infection with oncogenic human papillomavirus (HPV), mainly genotype 16, is a pre-requisite to the development of cancer [2]. In older, predominantly post-menopausal women, cancer arises in vulvar skin affected by squamous hyperplasia or lichen sclerosus [3].

Worldwide, the incidence of vulvar cancer ranges from 0.0 to 3.5 per 100,000 [4]. Although relatively uncommon, the incidence is increasing, largely in younger women, associated with oncogenic HPV and smoking [5-7]. In women aged less than 50 years, the highest incidence rate has been reported in the Northern Territory of Australia (2.3 per 100,000) [4]. This is largely the result of a very high incidence in young Aboriginal (Indigenous) women living in a distinct geographic region known as Arnhem Land.

Between 1996–2005, in the Arnhem Land region, the incidence of vulvar cancer among Indigenous women aged less than 50 years was 31.1 per 100,000 (95% Confidence Intervals (CI) 13.1-49.1), over 50 times higher than the rate for the total Australian population of the same age-group [8]. An excess of high-grade vulvar intraepithelial neoplasia (VIN grade 2/3), the precursor lesion to vulvar cancer also occurs in this population [8].

The cause of this very high incidence is unclear. The cases are almost entirely restricted to the under 50 age-group, suggesting that excess oncogenic HPV infection is a key etiological factor. The prevalence of vulvar HPV infection in this population and in other Australian women is unknown. We undertook a cross-sectional study to assess prevalence of oncogenic anogenital HPV infection in Indigenous women residing in the Arnhem Land region. Samples were collected from the cervix and vulvar/vaginal/perianal area. The aims of this study were two-fold. First, to compare the vulvar/vaginal/perianal and cervical HPV prevalence in Arnhem Land women. Second, to compare the prevalence of cervical HPV in this population to that of other Australian women, to provide an indirect indication of whether oncogenic vulvar HPV infection is more common in the Arnhem land region.

## Methods

### Participants and procedures

A cross-sectional survey was undertaken in 2007–2009 in 11 primary health centres providing services to 9 Indigenous communities and a number of smaller outstations in the Arnhem Land region. The characteristics of this region have been described previously [8]. Briefly, this is a sparsely populated area in the northern part of Australia. The majority of the population are Indigenous and living in discrete remote communities of varying population size ranging from several hundred to approximately 2,500 people. Health centres based in the communities provide primary health care and limited access to visiting specialist medical services.

Communities were identified based on our earlier work [8] and represented the majority in the region with vulvar cancer cases. The study was preceded by an extensive period of consultation, which included holding community forums to raise awareness of vulvar cancer. During this time an Indigenous reference group comprising Indigenous women from the region was established to advise on all aspects of the study.

Women were eligible to participate if they met the following criteria: Aboriginal and/or Torres Strait Islander, aged between 18 and 60 years, and their usual place of residence was in a community in the Arnhem Land region.

There were four methods of recruitment: (1) approaching women due for Pap screening based on health centre recall systems; (2) holding community forums; (3) holding “women’s health weeks” which had a focus on health education and screening; and (4) approaching women who presented to the health centre when the research team were present.

All participants gave written informed consent, and the study procedures conformed to the principles of the Declaration of Helsinki. Consenting women were invited to have a Pap test, specimen collection for combined vulvar/vaginal/perianal (VVP) and separate cervical HPV detection and genotyping, swabs and collection of blood (where indicated) for assessment of other sexually transmissible infections (STIs), and clinical examination for vulvar lesions. Samples for VVP genotyping were collected by clinical staff performing a single sweep swab of the vulva, vagina and peri-anal region using an established protocol [9]. The VVP swab was undertaken before the Pap test. Samples for endocervical HPV genotyping were taken from the PreservCyt endocervical sample collected during Pap screening. The endocervical sample was collected prior to any other swabs of the cervix or posterior fornix, using an endocervical brush that was removed carefully to avoid vaginal contamination. The majority (75%) of specimens were collected by the same clinician (MN).

Women with clinical signs of anogenital disease were referred to a gynecologist for colposcopy and biopsy for definitive histological diagnosis and treatment. Abnormal Pap and STI test results were managed by the health centre in accordance with local guidelines. Women testing positive for high-risk (HR) HPV but with a normal Pap test result were offered a repeat Pap test and HPV genotyping at 12 months.

### HPV DNA testing

For cervical and VVP specimens, cellular and viral DNA was extracted using the MagNA Pure LC (MP) isolation and purification system (Roche Molecular Systems Alameda, CA, USA) with a modified protocol [10]. For cervical specimens, HR-HPV was detected using the Amplicor HPV test (Roche Molecular System) targeting 165 bp of the L1 gene of the 13 HR-HPV anogenital types (16, 18, 31, 33, 35, 39, 45, 51, 52, 56, 58, 59 and 68). Sample adequacy was determined as the Amplicor PCR method allows for simultaneous amplification of ~265 bp region of human ß-globin.

Any samples negative for HR-HPV on the Amplicor HPV test were further assessed for the presence of HR- and low risk (LR)-HPV. Briefly, 20 uL of extracted DNA was amplified for 40 cycles using 50 rmol of each of the Ll consensus primers PGMY09/11 [11,12]. Amplification products were hybridised with a biotin-labelled HPV Ll generic probe [13] and captured on streptavidin-coated plates (Roche Biochemicals) [14]. The bound hybrid was detected by an anti-digoxigenin peroxidase conjugate by use of the colourimetic substrate ABTS [14].

### HPV DNA genotyping

Any sample positive for HR- or LR-HPV DNA was genotyped on the Roche Linear Array (LA) HPV genotyping test (Roche Molecular Systems). This test directs amplification of a 450 bp region of the HPV L1 gene and allows identification of 37 anogenital HPV genotypes (6, 11, 16, 18, 26, 31, 33, 35, 39, 40, 42, 45, 51, 52, 53, 54, 55, 56, 58, 59, 61, 62, 64, 66, 67, 68, 69, 70, 71, 72, 73, 81, 82, 83, 84, IS39 and CP6108) as well as amplification of 265 bp region of the human ß-globin gene, serving as an internal control. Samples were denatured and detected using a modified method as previously described [15,16]. Due to possible cross-reactivity of the HPV-52 probe with types 33, 35, and 58 amplicons, samples positive for the HPV-52 probe alone were classified as HPV-52 positive, whilst those reactive with this probe and one or more of HPV-33, 35, and 58 probes were further tested using a real-time PCR assay with an HPV-52 specific hydrolysis probe [17], allowing detection of HPV-52 DNA in the presence/absence of other genotypes.

At the completion of the study any cervical or VVP sample that was positive on any test had the corresponding cervical or VVP sample genotyped to ensure correct comparison of differing samples. There were 10 cervical samples and 5 VVP samples that were positive on initial HPV DNA testing, but no HPV genotype was detected on LA.

### Primary outcomes

The primary outcomes were the cervical and VVP prevalence of HR-HPV, defined as detection of any one of the following HPV types: 16, 18, 31, 33, 35, 39, 45, 51, 52, 56, 58 or 59). Secondary outcomes included the cervical and VVP prevalence of: probable HR-HPV (type 68), possible HR-HPV (types 26, 53, 66, 67, 69, 70, 73 or 82), any-HPV (presence of any of the 37 types detectable on LA), LR-HPV (types 6, 11, 40, 42, 54, 55, 61, 62, 64, 71, 72, 81, 83, 84, CP6108, IS39), multiple HR types, multiple LR types, individual type-specific HPV and vaccine preventable types (6, 11, 16, 18). All HPV prevalence figures were based on HPV as detected on LA. HPV types were classified into carcinogenic groups according to the International Agency for Research on Cancer [18].

### Analyses and sample size

Proportions and means/medians were calculated to summarize the data as appropriate. As there is no data on the prevalence of vulvar HPV infection in Australian women, we compared the prevalence of cervical HR-HPV infection in Arnhem Land women to that of other Australian women (Indigenous and non-Indigenous) based on the results of the Women's HPV prevalence Indigenous Non-Indigenous Urban Rural Study (WHINURS) [19]. This is a national study of cervical HPV genotype prevalence undertaken between 2005–08 by members of the research team (JRC, SNT, SMG) using the same methodology (sample collection and analysis) and the same laboratory for HPV genotyping as this study. The McNemar’s matched pairs test was used to assess whether the prevalence of VVP HPV was similar to the prevalence of cervical HPV infection, in women with both samples adequate for HPV analysis. This comparison was made to provide an indirect indication of whether VVP HPV infection is more common in Arnhem Land women than other Australian women. Chi-square analysis was undertaken to assess whether HPV prevalence varied across 10-year age bands. Where the expected cell counts were less than 5, the exact significance probability is reported. A P-value <0.05 was considered statistically significant. All analyses were conducted using Stata version 10.0 (Stata Corporation, College Station, TX, USA).

The sample size was estimated using preliminary data in 2006 from the WHINURS [19]. To detect a difference in the prevalence of HPV-16 and 18 combined of 14.5% for Indigenous women in Arnhem Land compared with the 9.5% found in the WHINURS, 521 women were required (alpha level 0.05, 80% power, one-sided test).

This study was approved by the Human Research Ethics Committee of the Menzies School of Health Research and the Northern Territory Department of Health and Community Services, and its Aboriginal subcommittee.

## Results

Five-hundred and sixty-two women consented to participate. Eleven women (1.9%) were found to be ineligible after enrolment due to age and were excluded. The characteristics of participants are summarised in Table 1. Fifty-three women (9.6%) had never previously had a Pap smear. Two women (0.4%) presented with current anogenital symptoms.

**Table 1:** Demographic characteristics, previous anogenital neoplastic lesions, and history of chronic disease among participating women

**Characteristics**	**N=551**	**%**
Mean age, years (SD)	34.5	10.7
Median BMI, kg/m^2^ (IQR)	22.8	19.2-26.9
Current smoker	368	66.8
On hormonal contraception	220	39.9
Any previous anogenital dysplastic/neoplastic lesion*	168	30.5
cervical low grade lesion	142	25.8
cervical high-grade lesion	75	13.6
invasive cervical cancer	1	0.2
vulvar intraepithelial neoplasia	3	0.5
invasive vulvar cancer	2	0.4
vaginal intraepithelial neoplasia or invasive vaginal cancer	0	0
anal intraepithelial neoplasia	0	0
invasive anal cancer	2	0.4
Vulvectomy	2	0.4
Hysterectomy	19	3.4
Diabetes	54	9.8
Chronic renal disease	38	6.9
Acute rheumatic fever	24	4.4
Asthma	22	4.0
Autoimmune disease	15	2.7
Chronic liver disease	4	0.7

Figures are n, % unless otherwise indicated.

*Histologically confirmed.

*SD* = standard deviation, *IQR* = interquartile range, *BMI*= body mass index.

All women consented to a vulvar examination and VVP swab collection. However, VVP samples were not adequate for HPV testing for 30 women, due to samples leaking during transportation. Five-hundred and nine women consented to Pap screening, and of these, cervical samples were not adequate for HPV testing in two women. A vault smear was undertaken in six women with a hysterectomy, and these samples were excluded from the cervical samples. As a result, a total of 521 VVP swabs and 501 cervical swabs were adequate for analysis.

On examination, one new case of vulvar cancer and two new cases of high-grade vulvar lesion were found; six definitive high-grade cervical lesions were detected on Pap cytology screening (Table 2). No new anal lesions were identified.

**Table 2:** Vulvar abnormalities, cervical cytology, and sexually transmissible infections detected at study examination

	**N=551**	**%**
Suspected vulvar abnormality	20	3.6
Confirmed vulvar abnormality	9	1.6
low grade VIN	3	0.5
high-grade VIN^*^	2	0.4
invasive vulvar cancer^*^	1	0.2
genital warts	1	0.2
other (e.g. trauma, non-specific inflammation)	3	0.5
Pap test performed	509	92.4
thin prep and/or slide	502	91.1
vault smear	7	1.3
Satisfactory Pap specimen^†^	501	98.4
Pap result normal^‡^	464	92.6
Abnormal Pap results^‡^	37	7.4
possible LGSIL	19	3.8
definite LGSIL	8	1.6
possible HGSIL	4	0.8
definite HGSIL	6	1.2
Sexually transmissible infections		
positive for *N. gonorrhoeae*^§^	19	3.9
positive for *C. trachomatis*^§^	25	5.1
positive for *T. vaginalis*^§^	132	26.9
positive for active syphilis (*T. pallidum*)^¶^	11	2.5
previous positive for past and/or treated syphilis^¶^	76	17.3
HIV^**^	0	0

^*^One woman had a high-grade and an invasive cancerous lesion on the vulva.

^†^Of n=509 Pap tests performed.

^‡^Of n=501 satisfactory Pap specimens.

^§^Of n=491 tests.

^¶^Of n=439 tests.

^**^Of n=428 tests.

*VIN* = vulvar intraepithelial neoplasia, *LGSIL* = low-grade squamous intraepithelial lesion, *HGSIL* = high-grade squamous intraepithelial lesion, *SCC* = squamous cell carcinoma, *HIV* = human immunodeficiency virus.

### HPV prevalence

The prevalence of VVP HR-HPV was 39% compared with the cervical HR-HPV prevalence of 26% (Table 3). Co-infection with multiple VVP HR-HPV types occurred in 15% of women, and 6% had multiple cervical HR-HPV types. HPV-16 was the most common genotype detected in both the cervix and the VVP area (Table 3). Of the vaccine preventable types, the prevalence of type 16 and/or 18 was 13% in the VVP area and 7% in the cervix; types 6, 11, 16 and/or 18 were in 15% of women in the VVP area and 8% in the cervix.

**Table 3:** Prevalence and distribution of HPV DNA detected in cervical and vulvar/vaginal/perianal samples (n=1022)

**HPV type**	**Cervical**		**Vulvar/vaginal/perianal**	
	**n=501**	**%**	**n=521**	**%**
HR types^*^	130	25.9	201	38.6
Multiple HR types	29	5.8	80	15.4
Probable HR type (genotype 68)^†^	8	1.6	14	2.7
Possible HR types^‡^	47	9.4	117	22.5
Multiple possible HR types^‡^	5	1.0	12	2.3
Any HPV	221	44.1	334	64.1
Multiple HPV types	81	16.2	209	40.1
Any LR types	108	21.6	213	40.9
Multiple LR types	23	4.6	89	17.1
Individual HR types^*^				
16	29	5.8	58	11.1
51	22	4.4	49	9.4
52	20	4.0	38	7.3
58	17	3.4	28	5.4
35	11	2.2	24	4.6
39	18	3.6	24	4.6
56	8	1.6	24	4.6
59	12	2.4	21	4.0
18	8	1.6	15	2.9
45	6	1.2	11	2.1
33	7	1.4	10	1.9
31	6	1.2	9	1.7
Individual possible HR types^‡^				
53	28	5.6	59	11.3
70	6	1.2	25	4.8
66	8	1.6	16	3.1
69	6	1.2	12	2.3
73	4	0.8	11	2.1
26	0	-	2	0.4
67	0	-	2	0.4
82	0	-	2	0.4
Individual LR types				
81	24	4.8	57	10.9
62	19	3.8	50	9.6
72	20	4.0	38	7.3
84	16	3.2	35	6.7
55	14	2.8	28	5.4
71	6	1.2	25	4.8
42	8	1.6	19	3.6
54	5	1.0	17	3.2
61	4	0.8	17	3.2
CP6108	6	1.2	16	3.1
IS39	2	0.4	9	1.7
83	7	1.4	8	1.5
6	5	1.0	6	1.2
40	4	0.8	5	1.0
11	0	-	4	0.8
64	0	-	0	-

^*^IARC Group 1, ^†^IARC Group 2A, ^‡^IARC Group 2B.

*HPV*=human papillomavirus, *HR*=high-risk, *IARC*=international agency for research on cancer, *LR*=low-risk.

Four hundred and seventy-two women had both samples (cervical and VVP) adequate for HPV analysis. The prevalence of HR-HPV was significantly higher in the VVP samples than the cervical samples (39% vs 26%, p<0.0001), as was the presence of multiple HR-HPV types (16% vs 6%, p<0.0001) and possible HR-HPV types (22% vs 10%, p<0.0001).Type-specific HPV prevalence was significantly higher in the VVP samples for the following HR-HPV types: 16 (11% vs 6%, p<0.0001), 51 (10% vs 4%, p<0.0001), 52 (8% vs 4%, p=0.002), 56 (5% vs 1%, p=0.0001) and 35 (5% vs 2%, p=0.004); and possible HR-HPV types: 53 (11% vs 6%, p<0.0001), 70 (4% vs 1%, p=0.0001) and 73 (2% vs 1%, p=0.03).

### Age-specific HR-HPV and HPV-16 prevalence

**Figure 1:**
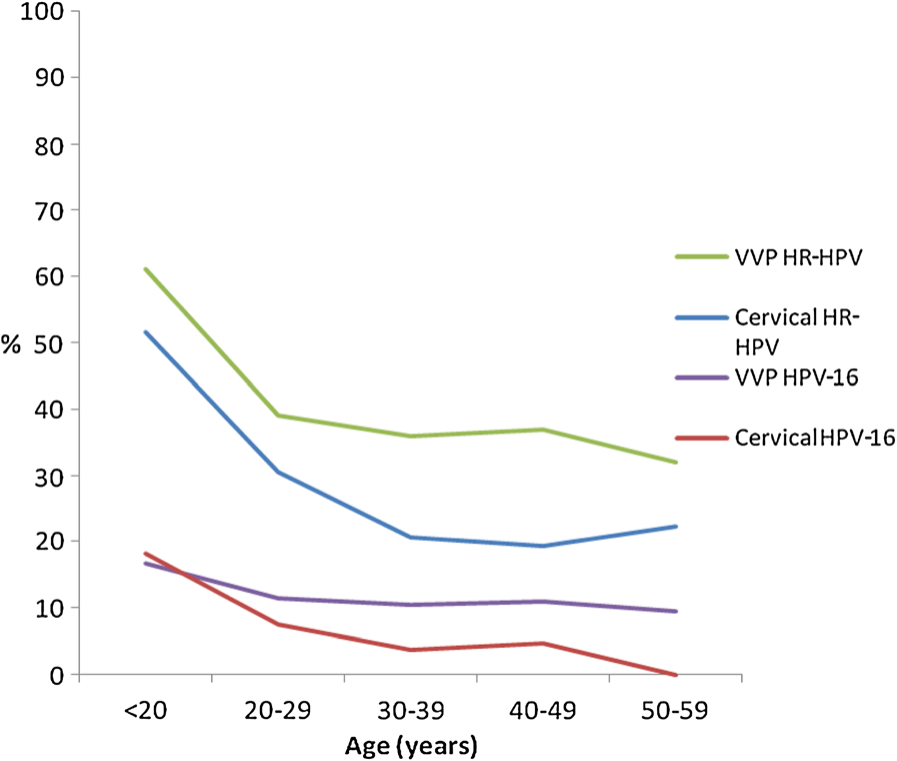
Age-specific prevalence of VVP and cervical HR-HPV and HPV-16.

For both VVP and cervical infections, HR-HPV prevalence peaked in the age-group <20 years and declined thereafter (Figure 1). There was a significant difference in the age distribution of cervical HR-HPV (p=0.001); the difference in age distribution of VVP HR-HPV was of borderline statistical significance (p=0.06). A different pattern was observed for the age-specific prevalence of HPV-16 infection. There was a marked and significant decline in prevalence of cervical HPV-16 infection with age (p=0.007), whereas VVP HPV-16 infection declined initially after the <20 age-group and then remained constant across the older age-groups (p=0.835) (Figure 1).

## Discussion

In this community-based study of Indigenous women in the Arnhem Land region, where there is a very high incidence of vulvar neoplasia [8], genital HPV infection was common, and significantly more prevalent and more diverse in the VVP area than the cervix. HPV-16 was the most common genotype detected in both sites, and in general the type-specific prevalence of HPV infection was significantly higher in the VVP area relative to the cervix for both oncogenic and LR-HPV types.

Just over a quarter of all women in this study had a cervical HR-HPV infection, which is very similar to the prevalence found in the WHINURS, the largest examination of cervical HPV genotype prevalence in Australian women (cervical HR-HPV prevalence 30.0% in non-Indigenous women and 31.3% in Indigenous women aged <41 years) [19]. In our study population, we found a significantly higher prevalence of HR-HPV infection in the VVP area than the cervix. These findings suggest that there is no excess prevalence of oncogenic cervical infection amongst Indigenous women in Arnhem Land. Vulvar/vaginal/perianal infection was not assessed in the WHINURS.

A higher rate of HPV detection in vulvar/vaginal samples than cervical samples has been reported previously [20,21]: however, the discrepancy between anogenital sites found in this study is substantially higher than in previous studies. We found the prevalence of any HPV and HR-HPV in the VVP area was 1.5 times higher than in the cervix (absolute differences 20% and 13%, respectively), whereas previous studies report a higher prevalence in vulvar/vaginal samples than cervical in the order of 1.2 times for any HPV and 1.1 times for HR-HPV (absolute differences ranging from 2-6% and 1-5%, respectively) [20,21], or no difference between genital sites [22-24].

The higher prevalence of VVP infection may reflect increased persistence of infection or continuing re-infection with different genotypes. Our finding that VVP HPV-16 prevalence did not vary significantly by age, whereas there was a marked and significant decline in cervical HPV-16 prevalence, suggests that increased persistence of HR types on the vulva alone may be occurring. This may explain why the cervical cancer incidence in this population is no higher than in other Indigenous women in the Northern Territory [25]. We are not aware of any other reports of increased persistent infection in other genital sites than the cervix, although increased persistence in the anus relative to the cervix has been reported in immunocompromised women [26].

The higher prevalence of infection in the VVP area relative to the cervix could also reflect incident infections that have not yet ascended to the cervix. Winer et al. [27] demonstrated a shorter interval between report of a new sex partner and HPV infection for genotypes on the vulva/vagina than cervix. This is unlikely to completely explain the higher prevalence seen in this study as a subsequent report by the same group found the majority of incident infections were still detected simultaneously in both sites [28]. Also, the relatively larger surface area swabbed for the VVP sample than the cervix may have resulted in enhanced detection of HPV [29]. Although if this were the primary reason for the higher prevalence, a smaller difference between sites consistent with previous studies of genital HPV would be expected [20,21]. The higher prevalence observed in the VVP sample could also reflect contamination from or oversampling of the perianal region, as a higher prevalence of anal HPV than cervical has been reported in high-risk women [30].

Longitudinal data are needed to examine persistence of VVP HPV infection in this population. However, while speculative, neither a 1.5-fold higher prevalence nor increased persistence of HR-HPV types is likely to be sufficient to explain the fifty-times higher incidence of vulvar cancer in this population compared to other Australian women. Other factors that alter the balance between HPV infection and host immunity are likely to be present. Such factors could be genetic, affecting the susceptibility to or inability to clear HPV, or environmental, possibly altering the natural history of infection of the vulvar epithelium. An interaction between a combination of risk factors (e.g. genetic, HPV, smoking) is also possible; genetic variation in Th1 cytokines has been shown to modify the risk of vulvar cancer among smokers [31]. A very high rate of smoking was found in this study (67%), consistent with previous data from the Northern Territory [32]. Further research is currently underway examining possible environmental and genetic factors in this population.

There are several limitations of this study. There is no data on HPV prevalence in the VVP area in other Australian women, and so a direct comparison of VVP HPV prevalence was not possible. The study design was cross-sectional, therefore persistence of HPV infection could not be adequately assessed. Similarly, detection of HPV DNA only reflects current infection or carriage status, hence there is no information about cumulative lifetime exposure to HPV. Some women (7%) did not consent to have a Pap smear, which reduced the number of women with both samples available for analysis. There was no difference in demographic characteristics or history of anogenital lesions between women who consented to a Pap smear and those who did not, suggesting that this was not a major source of bias. Finally, our results will not generalize to other Indigenous or non-Indigenous women, as the study was purposely designed to examine the prevalence of HPV in a population with, to our knowledge, the highest incidence of vulvar cancer reported worldwide.

## Conclusions

In young Indigenous women in the Arnhem Land region of Australia, a population subject to a cluster of vulvar cancer, we have shown that the prevalence of cervical HPV infection is similar to other Australian women, both Indigenous and non-Indigenous. We found a significantly higher prevalence of vulvar/vaginal/perianal infection to cervical in Arnhem Land Indigenous women. Although in other Australian women the prevalence of HPV infection in the vulvar/vaginal/perianal area is unknown, in our study population, the higher prevalence of infection in this area relative to cervical is unlikely to completely explain this cancer cluster. Further investigation of possible genetic and/or environmental risk factors that may impair host immunity is required.

## Abbreviations

BMI: Body mass index; DNA: Deoxyribonucleic acid; HGSIL: High–grade squamous intraepithelial lesion; HIV: Human immunodeficiency virus; HPV: Human papillomavirus; HR: High-risk; HR-HPV: High-risk human papillomavirus; IARC: International agency for research on cancer; IQR: Interquartile range; LGSIL: Low-grade squamous intraepithelial lesion; LR: Low-risk; SCC: Squamous cell carcinoma; SD: Standard deviation; STI: Sexually transmissible infection; VIN: Vulvar intraepithelial neoplasia; VVP: Vulvar/vaginal/perianal; WHINURS: Women's HPV prevalence indigenous non-indigenous urban rural study.

## Competing interests

SMG has received advisory board fees and grant support from CSL and GSK; lecture fees from Merck, GSK and Sanofi Pasteur; funding (through her employing institution) to conduct HPV vaccine studies for MSD and GSK; and is a member of the Merck Global Advisory Board and the Merck Scientific Advisory Committee for HPV. SET is the recipient of a GlaxoSmithKline Australian Postgraduate Support Grant for work outside of this submitted manuscript. CSL Biotherapies have provided funding to the authorship group to support a workshop on genetic susceptibility to vulvar cancer.

## Authors’ contributions

JRC, ARR, SNT and SMG had a primary role in developing the study design; DT and MN were responsible for participant recruitment, sample collection and data management; SET undertook the HPV genotyping of samples and all other associated laboratory work under the supervision of SNT. ARR conducted the analyses and drafted the manuscript; JRC, SET, DT, MN, MJD, MMO, CMC, IZ, JM and SMG contributed to the interpretation of analyses and assisted with preparation and editing of the manuscript. All authors read and approved the final manuscript.

## Funding

This work was supported by the National Health and Medical Research Council (NHMRC) in Australia, Grant ID: 436013. The views expressed in this publication are those of the authors and do not reflect the views of NHMRC. Alice Rumbold is supported by the Jean B Reid Fellowship from the University of Adelaide Medical Endowment Funds. Sarah Tan is supported by the Royal Women’s Hospital Post Graduate Degree Scholarship from the Royal Women’s Hospital, Melbourne Australia.

## Pre-publication history

The pre-publication history for this paper can be accessed here:

http://www.biomedcentral.com/1471-2334/12/243/prepub
